# Radiofrequency Microneedling-Mediated Enhancement in Percutaneous Delivery of Topical Triamcinolone Acetonide: An In Vivo Quantitative Study

**DOI:** 10.3390/life16071074

**Published:** 2026-06-27

**Authors:** Rosalyn Kupwiwat, Rungsima Wanitphakdeedecha, Teerapat Wannawittayapa, Panyapat Buranaporn, Poonsin Poungpairoj, Theeraporn Maneesawat, Varangkana Tantithavorna, Premrutai Thitilertdecha

**Affiliations:** 1 Department of Dermatology, Faculty of Medicine Siriraj Hospital, Mahidol University, 2 Wanglang Road, Siriraj, Bangkok Noi, Bangkok 10700, Thailand; 2 Siriraj Research Group in Immunobiology and Therapeutic Sciences, Research Department, Faculty of Medicine Siriraj Hospital, Mahidol University, 2 Wanglang Road, Siriraj, Bangkok Noi, Bangkok 10700, Thailand

**Keywords:** microneedling, radiofrequency, triamcinolone acetonide, dermal absorption, drug delivery

## Abstract

Background: The stratum corneum (SC) limits the efficacy of topical corticosteroids. Radiofrequency microneedling (RFMN) may enhance drug delivery; however, quantitative in vivo human data remain limited. Objective: To evaluate RFMN-assisted delivery of topical triamcinolone acetonide (TAC) and determine optimal device parameters. Methods: In this prospective intra-individual study, five healthy volunteers underwent TAC application followed by RFMN on volar forearms. Two insertion depths (0.5 and 2.5 mm) and three RF energy levels (0, 390, and 750 mJ) were assessed. TAC uptake into the SC was quantified after 6 h using tape stripping and UPLC-PDA. Results: RFMN significantly enhanced TAC absorption compared with topical application alone. The highest uptake was observed at 2.5 mm insertion depth without RF energy (3.5-fold increase). At 0.5 mm depth, only moderate RF energy (390 mJ) improved penetration (2.3-fold), whereas higher energy showed no benefit. TAC distribution profiles demonstrated increased drug deposition primarily in superficial SC layers. Conclusions: Insertion depth is the primary determinant of RFMN-assisted drug delivery, while RF energy plays a secondary, context-dependent role. RFMN represents a promising strategy to enhance topical corticosteroid delivery with potential clinical applications in inflammatory dermatoses and scar modulation.

## 1. Introduction

Topical medications are widely used in the treatment of skin diseases; however, their absorption into the skin is usually limited by the skin barrier, particularly the stratum corneum (SC), resulting in insufficient drug concentrations at target sites (i.e., viable epidermis and dermis) for therapeutic efficacy. To achieve effective therapeutic dose, the active drug must cross the SC barrier [1,2]. The stratum corneum was selected as the target analytical compartment because it represents the principal permeability barrier regulating topical drug delivery and serves as the initial reservoir influencing subsequent passive diffusion into deeper skin layers. Triamcinolone acetonide (TAC) is one of the most commonly prescribed corticosteroids for various dermatologic conditions, such as hypertrophic scars, keloids and atopic dermatitis. Nevertheless, its percutaneous absorption is poor in terms of rate and extent [3]. Topical bioavailability of TAC was also not improved by increasing skin hydration through pre-occlusion but increased just twice when occlusion was applied after application [4]. Moreover, the extent of TAC in the SC did not depend on the dose applied and application frequency [5].

Despite their widespread clinical utility, prolonged or inappropriate use of topical corticosteroids is associated with numerous local and systemic adverse effects. The most common cutaneous complications include skin atrophy, striae, telangiectasia, purpura, barrier dysfunction, delayed wound healing, steroid-induced acne and rosacea, as well as increased susceptibility to cutaneous infections. These adverse ef-fects are primarily attributed to corticosteroid-induced suppression of fibroblast activ-ity, reduced collagen synthesis, and impaired epidermal barrier homeostasis. In addi-tion, prolonged exposure may rarely result in systemic complications, including hypo-thalamic–pituitary–adrenal (HPA) axis suppression, hyperglycemia, and Cushing syndrome, particularly when high-potency corticosteroids are applied over large sur-face areas or under conditions that enhance percutaneous absorption [6,7].

Therefore, strategies that improve targeted cutaneous delivery while reducing the required corticosteroid dose and unnecessary prolonged exposure are of considerable clinical interest. Microneedle-assisted drug delivery has emerged as a promising ap-proach by transiently bypassing the stratum corneum barrier and enhancing localized drug deposition within the skin. By increasing delivery efficiency, this technique may potentially achieve improved local therapeutic effects with lower drug exposure, thereby reducing the risk of treatment-related complications.

Device-assisted drug delivery using mechanical disruption and energy-based modalities has been introduced to overcome the epidermal barrier by bypassing the SC. They create microchannels or microthermal treatment zones that act as reservoirs for topical drug molecules [8,9,10]. Multiple strategies have been explored to overcome stratum corneum barrier limitations, including chemical penetration enhancers, iontophoresis, microneedling, laser-assisted drug delivery, nanoparticle-based formulations, and particulate delivery systems. These approaches vary in their mechanisms, efficiency, cost, and clinical applicability. Microneedle-assisted iontophoresis, for example, combines physical barrier disruption with electrically driven transport and has demonstrated enhanced transcutaneous delivery of anti-inflammatory compounds [11]. Nevertheless, quantitative in vivo human data directly characterizing parameter-dependent enhancement mechanisms remain limited.

Different device technology and parameters affect cutaneous channel properties including depth, density, width and coagulation zone formation, thereby influencing the degree of enhancement in cutaneous absorption [12,13,14,15,16]. Radiofrequency microneedling (RFMN) is a minimally invasive technique that involves the creation of microchannels at controlled depths via microneedles in a combination with radiofrequency (RF) energy delivery. Thermal deposition occurs at the needle tips in insulated configurations, whereas non-insulated needles deliver the energy along the full length of the needle shaft. Unlike lasers which dictate the depth of microchannels via pulse parameters, RF delivers oscillating electrical current to generate thermal energy and subsequently induces coagulation [17]. Regarding drug delivery enhancement, RFMN has been proved to promote the cutaneous absorption of topical active compounds in skin and hair follicle related diseases, such as periorbital wrinkles, acne, atrophic acne scars, melasma and alopecia as well as skin rejuvenation [18,19,20,21,22,23]. However, these existing studies have focused on hydrophilic drugs and have relied largely on in vitro and ex vivo assessments using excised animal or human skin along with observed clinical improvements rather than quantitative in vivo studies in humans.

Among commercially available RFMN systems, only the new generation POTENZA™ RFMN features a fusion tip-an insulated dual air-chamber pumping tip designed to facilitate the dermal drug penetration and potentially improve bioavailability with the minimal thermal damage to the epidermis. This system also operates using monopolar radiofrequency in which the energy is dispersed and transmitted more deeply across the skin [24]. It is recommended to perform RFMN immediately after topical drug application rather than using RFMN as a pretreatment. Nevertheless, despite being specifically designed to enhance drug delivery, there is currently a lack of quantitative evidence supporting its efficacy. This study thus aimed to evaluate the impact of the RFMN device (POTENZA™) on the cutaneous TAC absorption in vivo in healthy volunteers using non-invasive tape stripping and quantitative techniques as well as to determine the optimal insertion depth and RF energy parameters. The safety of the RFMN treatment was also observed using visual analog scale (VAS) pain score along the experiment periods and 1-week follow-up. Therefore, we hypothesized that RFMN enhances topical TAC delivery in a parameter-dependent manner, with insertion depth and RF energy exerting distinct mechanistic effects on drug penetration.

## 2. Materials and Methods

### 2.1. Study Design

This prospective, randomized, intra-individual pilot study evaluated RFMN-assisted delivery of topical TAC in healthy volar forearm skin. The protocol was approved by the Siriraj Institutional Review Board (SIRB) of the Faculty of Medicine Siriraj Hospital at Mahidol University, Thailand [COA no. Si 807/2023]. The study was conducted in accordance with the ethical principles of the Declaration of Helsinki and its subsequent amendments. The clinical trial was registered with the Thai Clinical Trials Registry (TCTR20250318001).

### 2.2. Human Subjects

Five healthy volunteers aged between 28–48 years, with clinically normal volar forearm skin and without tattoos, scarring, sunburn on the volar forearm were recruited into the study. Sample size determination was performed in consultation with a biostatistician. Given the absence of prior quantitative in vivo human studies evaluating percutaneous triamcinolone acetonide (TAC) delivery following radiofrequency microneedling (RFMN) treatment, formal power calculation based on effect size estimation was not feasible. Therefore, this investigation was designed as a pilot mechanistic study to establish preliminary quantitative evidence and generate effect size estimates for future confirmatory studies. All participants were confirmed to be non-pregnant and non-lactating. They had no active dermatitis, cutaneous infection, history of skin diseases, corticosteroid hypersensitivity, photosensitivity disorders, hypertrophic scars/keloids, visible skin abnormalities, prior skin or wound healing treatment and habitual smoking in the preceding 4 weeks. Written informed consent was obtained from all individuals prior to their enrollment in the study.

### 2.3. Topical Formulation and Microneedling-Assisted Drug Delivery

A commercially available triamcinolone acetonide formulation (Kanolone^®^, LBS Laboratory Ltd., Bangkok, Thailand), containing triamcinolone acetonide 10 mg/mL in suspension form, was selected based on prior published transdermal permeation studies and clinical relevance [25]. A minimally invasive radiofrequency microneedling device (POTENZA™, Cynosure, MA, USA) operating at monopolar 1 MHz radiofrequency and equipped with fusion tips was used. A single-use tip consisted of 21 microneedle electrodes with a diameter of 250 µm. Treatments were performed by a single practitioner using a single pass per treatment zone (≤10% overlap) at two needle depths (D; 0.5 and 2.5 mm). RF energy settings for each needle depth included 0 (off), level 2 (390 mJ), and level 5 (750 mJ). The shallow-est (0.5 mm) and deepest (2.5 mm) insertion depths available on the POTENZA™ sys-tem were selected to evaluate the influence of minimal and maximal mechanical dis-ruption on drug delivery enhancement. A full evaluation of all available insertion depths was not feasible due to the large number of possible treatment combinations and the exploratory nature of this pilot study. Therefore, representative extreme set-tings were selected to maximize the ability to detect differences in mechanical contri-bution to drug permeation. RF energy levels of 390 mJ and 750 mJ were chosen to rep-resent clinically relevant moderate and higher thermal exposure settings while main-taining procedural safety. The lowest RF setting (RF off) was included to isolate the ef-fect of mechanical microporation alone, whereas RF energies of 390 mJ and 750 mJ were selected to represent clinically relevant moderate and high thermal exposure set-tings, respectively. The 750 mJ setting already represents a relatively high RF exposure within routine clinical practice. Excessively higher RF energies were not investigated, as they may lead to unnecessary tissue coagulation, increased thermal injury, and treatment-related adverse effects without clear additional clinical benefit.

### 2.4. Skin Penetration Study

The experimental schedule was distributed across two consecutive days because the time-intensive protocol permitted completion of four treatment sites per day while minimizing participant burden and maintaining comparable skin conditions across treatment sessions. Each forearm was randomly assigned for 4 treatment sites. Randomization sequences were generated using a computer-based program (http://www.randomization.com, accessed on 1 October 2023). Eight skin sites were designated for 6 TAC combined with RFMN treatment groups: (i) D 2.5 mm, RF 0, (ii) D 2.5 mm, RF 2, (iii) D 2.5 mm, RF 5, (iv) D 0.5 mm, RF 0, (v) D 0.5 mm, RF 2, and (vi) D 0.5 mm, RF 5. These were compared with 2 control groups: (vii) RFMN only (D 2.5 mm, RF 0) without TAC application and (viii) TAC application only without RFMN. The treatment site was demarcated at 6 cm^2^ in area by a rectangular self-adhesive foam frame. One tape strip (Permacel J-LAR^®^) was discarded to remove stratum disjunctum before TAC application (100 µL) with and without RFMN assistance. All sites were occluded and left for 6 h before drug removal and tape stripping. Volunteers were asked to rate their pain using the VAS pain score (0 = no pain to 10 = extreme pain) throughout the experimental period and at the 1-week follow-up after a 2-day penetration experiment.

### 2.5. Tape Stripping and Tape Extraction

After the 6 h application period, the excess TAC solution was gently wiped away, and a new thin foam frame (3 cm^2^ in area) was placed at the same position as the original frame. An initial value of transepidermal water loss (TEWL) was measured using an AquaFlux^®^ evaporimeter (Biox System Ltd., London, UK). An adhesive tape strip (2.5 × 2.5 cm, Permacel J-LAR^®^, Pleasant Prairie, WI, USA) was subsequently applied to the skin, pressed firmly, and quickly removed. TEWL was then measured again. This procedure was repeated until the TEWL value reached 4 times the initial value or until 30 tape strips had been collected. The mass of skin removed on each tape was determined by measuring the difference in tape weight before and after tape stripping using an ultramicrobalance (CUBIC ultramicrobalance model MSA2.7S-000-DF, Sartorius, Göttingen, Germany). Together with the TEWL data, they were used to calculate the SC thickness of individual subjects [26]. The collected tapes were subsequently grouped for methanol extraction. The first and second tapes were analyzed individually and extracted with 1 mL of methanol, whereas the remaining tapes were combined into groups of 2–3 tapes and extracted with 1.5 mL of methanol. The samples were placed on a basic shaker overnight, after which the extracted solutions were collected and filtered through a 0.45 µm nylon membrane prior to TAC quantification. This in vivo methodology was previously proved to be reproducible and robust [27].

### 2.6. TAC Quantification

An ultra-performance liquid chromatography coupled with photodiode array (UPLC-PDA) method was used to quantify TAC amounts. The measurement was performed on a Waters Acquity™ UPLC system (Waters Corporation, Milford, MA, USA) and an Acquity UPLC™ BEH Shield RP18 column (1.7 µm, 100 mm × 2.1 mm i.d., Waters Corporation, Milford, MA, USA). The mobile phase was delivered at a flow rate of 0.45 mL/min and the column temperature was maintained at 50 °C throughout the analysis. The mobile phase consisted of acetonitrile (A) and 0.05% aqueous trifluoroacetic acid (*v*/*v*, B) and was operated under a gradient system of 30% A, 70% B for 3 min, 35% A, 65% B for 1 min and 30% A, 70% B for 1 min. TAC was detected at a wavelength of 242 nm using an Acquity UPLC^®^ PDA detector (Waters Corporation, Milford, MA, USA). All data were processed using the Empower 2 software (Waters Corporation, Milford, MA, USA). The complete validation of this analytical method was previously established [28].

### 2.7. Data Analyses

The primary outcome was cumulative TAC amount (µg/cm^2^) in the SC. Secondary outcomes included percentage TAC uptake relative to loading dose and TAC distribution across the SC depth. All statistical analyses were performed using GraphPad Prism^®^ version 11.0.0 (GraphPad Software, Inc., La Jolla, CA, USA). Data are presented as the mean or mean ± standard deviation (SD). Paired *t*-tests were used to compare treatment conditions. A *p*-value ≤ 0.05 was considered statistically significant. Normality was assessed using the Shapiro–Wilk test prior to parametric analysis. Given the intra-individual repeated-measures design, paired comparisons were selected to account for within-subject variability, allowing each participant to serve as their own control and improving sensitivity for detecting treatment-related differences.

## 3. Results

### 3.1. Demographics of Volunteers

A total of 5 healthy volunteers aged between 28 and 48 years were recruited in the study. The majority was female with Fitzpatrick skin type IV and all volunteers had similar demographic data as presented in Table 1. Although the study population comprised both genders, with known variations in keratinocyte size, skin pore size, and skin pH, transdermal drug permeability and overall skin barrier integrity have been reported to remain comparable [29]. None of the participants dropped out during the 2-day experimental period.

### 3.2. Impact of Needle Depth and RF Energy on TAC Absorption

**Figure 1:**
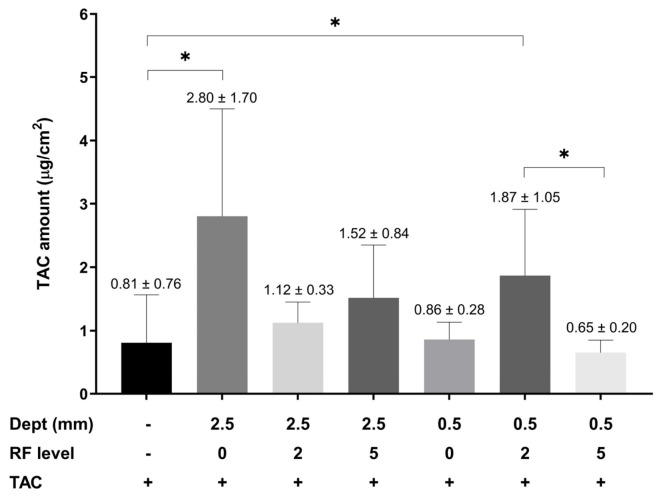
Total amounts of TAC in the SC after a 6−h application of a 10 mg/mL TAC solution with and without RFMN at different settings. For the RFMN-assisted groups, needle depths (D) of 2.5 and 0.5 mm and RF energy levels of 0 (off), 2 (390 mJ) and 5 (750 mJ) were applied. All data are presented as mean ± SD. * Indicates a statistically significant difference (*p* < 0.05).

TAC absorption without RFMN was low (0.81 ± 0.76 µg/cm^2^). RFMN significantly enhanced drug uptake under specific parameter settings. The highest absorption was observed at 2.5 mm depth without RF (2.80 ± 1.70 µg/cm^2^), representing a 3.5-fold increase compared with TAC alone (*p* = 0.0178). At 0.5 mm depth, only RF level 2 (390 mJ) significantly increased TAC uptake (2.3-fold, *p* = 0.0284), whereas higher RF energy showed no improvement. No detectable TAC was observed in control sites without drug application (Figure 1).

**Figure 2:**
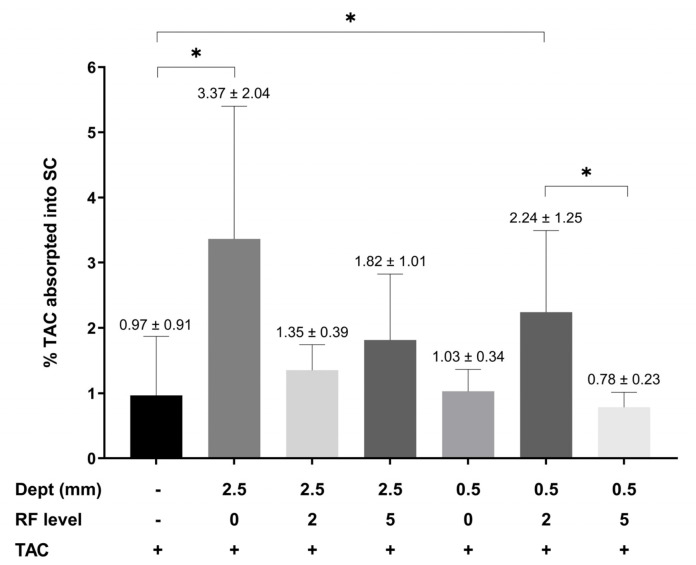
Percentages of TAC absorption in the SC after a 6−h application of a 10 mg/mL TAC solution with and without RFMN at different settings. For the RFMN-assisted groups, needle depths (D) of 2.5 and 0.5 mm and RF energy levels of 0 (off), 2 (390 mJ) and 5 (750 mJ) were applied. All data are presented as mean ± SD. * Indicates a statistically significant difference (*p* < 0.05).

Percentages of TAC permeated into the SC relative to the loading dose were calculated and are presented in Figure 2. Similar to the results of cumulative TAC amounts, only 0.97 ± 0.91% of TAC penetrated into the SC without RFMN. RFMN significantly increased TAC permeation by 3.5-fold at D 2.5 mm + RF 0 (*p* = 0.0178) and by 2.3-fold at D 0.5 mm + RF 2 (*p* = 0.0284). At a needle depth of 0.5 mm, RF 2 yielded higher TAC absorption than RF 5 (2.9-fold, *p* = 0.0404), while no enhancement was observed under the other conditions.

**Figure 3:**
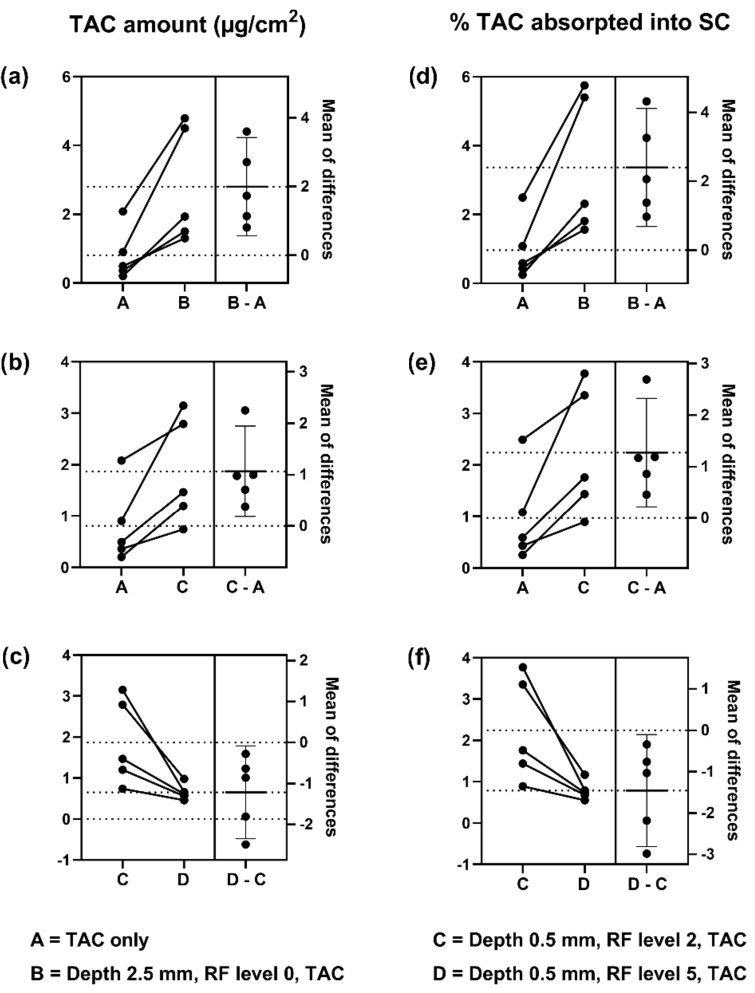
Estimation plots of TAC amount (µg/cm^2^) and percentage of TAC absorbed into the stratum corneum (% TAC uptake into SC) following a 6 h application of a 10 mg/mL TAC solution with and without radiofrequency microneedling (RFMN) under different treatment settings. For the RFMN-assisted groups, needle depths of 2.5 mm and 0.5 mm and RF energy levels of 0 (off), 2 (390 mJ), and 5 (750 mJ) were applied. The left panels display paired individual data distributions for each treatment condition, whereas the right panels show the mean differences with 95% confidence intervals. Panels (**a**,**d**) compare TAC only (A) with depth 2.5 mm, RF level 0, TAC (B); panels (**b**,**e**) compare TAC only (A) with depth 0.5 mm, RF level 2, TAC (C); and panels (**c**,**f**) compare depth 0.5 mm, RF level 2, TAC (C) with depth 0.5 mm, RF level 5, TAC (D).

To further confirm the enhancement of TAC absorption by RFMN despite the small sample size, estimation plots of both cumulative TAC amounts and percentage TAC uptake are presented in Figure 3. Variability between individual skin sites and the effect sizes between treatment settings with their corresponding 95% confidence intervals are illustrated. The RFMN settings of D 2.5 mm + RF 0 and D 0.5 mm + RF 2 consistently increased TAC absorption compared with TAC application alone (Figure 3a,b,d and e), as demonstrated by paired observations and positive mean differences (2.00 ± 1.15 µg/cm^2^, 1.06 ± 0.71 µg/cm^2^, 2.40 ± 1.38% and 1.27 ± 0.85%, respectively). In addition, the superiority of RF 2 over RF 5 at the same needle depth of 0.5 mm was observed with negative mean differences (Figure 3c,f; −1.22 ± 0.91 µg/cm^2^ and −1.46 ± 1.09%, respectively).

### 3.3. TAC Distribution Across the SC

**Figure 4:**
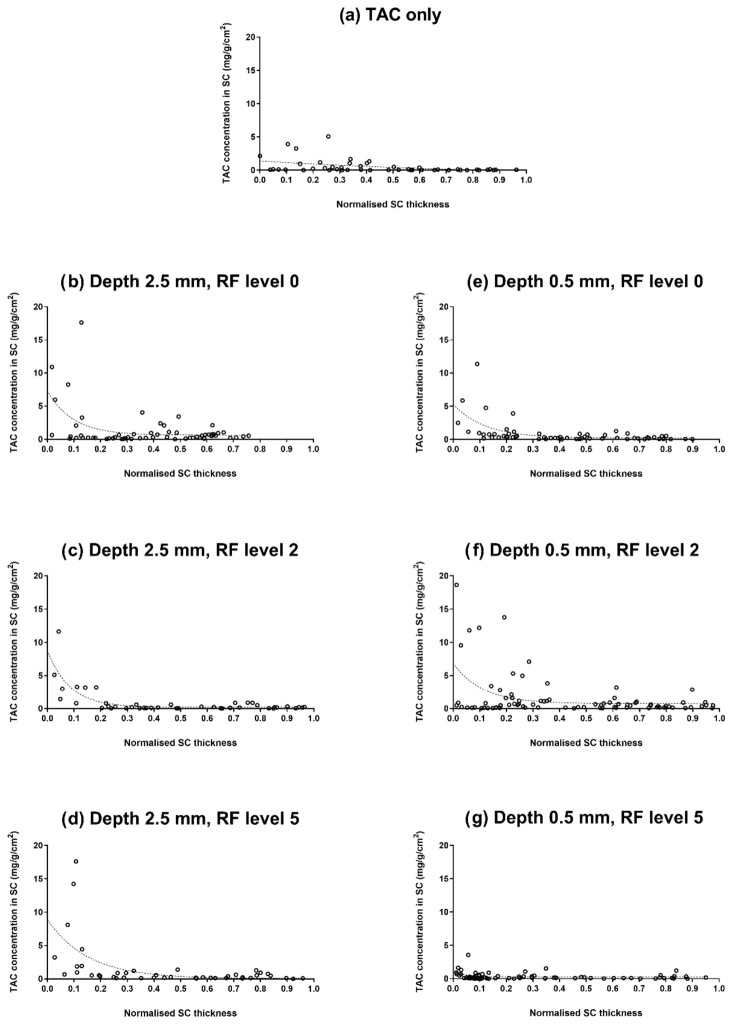
TAC concentration versus normalized stratum corneum (SC) thickness profiles following a 6 h application of a 10 mg/mL TAC solution with and without radiofrequency microneedling (RFMN) under different treatment settings. For the RFMN-assisted groups, needle depths of 2.5 mm and 0.5 mm and RF energy levels of 0 (off), 2 (390 mJ), and 5 (750 mJ) were applied. Panels represent the following treatment conditions: (**a**) TAC only; (**b**) depth 2.5 mm, RF level 0; (**c**) depth 2.5 mm, RF level 2; (**d**) depth 2.5 mm, RF level 5; (**e**) depth 0.5 mm, RF level 0; (**f**) depth 0.5 mm, RF level 2; and (**g**) depth 0.5 mm, RF level 5. Each point represents the TAC concentration measured within an individual tape strip layer normalized to SC thickness. Dotted lines indicate the fitted concentration–depth trend curves for each treatment condition.

The distribution profiles of TAC across the SC from the outermost to the innermost layers are presented as TAC concentration versus normalized SC depth (Figure 4). When TAC was applied alone, the drug concentration at the outermost layer was low (1.40 mg/g/cm^2^) and gradually decreased over the increasing SC depth. When applying RFMN, drug penetration was enhanced, resulting in increased TAC concentrations at the outermost SC layer under most treatment settings. Only the condition D 0.5 mm + RF 5 showed no increase and remained at a similar level. Between the two needle depths, D 2.5 mm produced greater enhancement than D 0.5 mm, with increases of 5.2−6.4−fold and 3.8−4.9−fold, respectively, compared with TAC alone. Overall, a more rapid exponential decay in TAC concentration across the superficial SC layers was observed, followed by gradual passive diffusion into the deeper layers. Despite this, TAC levels in the inner SC layers were still higher than those observed without RFMN assistance.

### 3.4. Safety Assessment of RF Microneedling-Assisted TAC Delivery

Safety assessment included procedural pain evaluation using the visual analog scale (VAS), monitoring of treatment-related adverse events during and after the experimental period, and clinical assessment of wound healing at follow-up. Participants were specifically evaluated for erythema, crusting, infection, pigmentary changes, textural alterations, and scar formation. RFMN is commonly associated with mild erythema and transient crusting, particularly when combined with tape stripping. Procedural discomfort was minimal, with a mean VAS pain score of 1.4 (range 1–2 out of 10). No serious adverse events or complications were observed during or after the experiments. Complete wound healing occurred in all participants within one week, without infection, pigmentary alteration, textural change, or scar formation.

## 4. Discussion

This study provides the first in vivo quantitative evidence that RFMN enhances topical corticosteroid delivery in human skin. The findings demonstrate that insertion depth is the primary determinant of drug penetration, whereas RF energy plays a secondary and parameter-dependent role. Although RFMN has been explored for percutaneous drug absorption via microchannel formation and RF-induced effects and has already been implemented in clinical practice, most supporting evidence is indirect and relied on clinical-based assessments rather than quantitative analyses [18,19,20,21,22,23]. Quantitative data on topical drug bioavailability and its distribution within the skin, particularly from in vivo human studies, also remain absent. Therefore, this study demonstrates the first investigation confirming the efficacy of RFMN in enhancing a topical corticosteroid TAC delivery into and across the skin in healthy volunteers using quantitative methods including tape stripping and UPLC-PDA detection. Parameter settings were also optimized to achieve the optimal enhancement efficacy.

In drug delivery systems, physicochemical properties of compounds critically influence its permeability into and across the skin. TAC has physicochemical properties that are generally considered favorable for skin penetration including a hydrophobic structure, relatively low molecular weight (MW = 434.5 Da) and an intermediate octanol–water partition coefficient (log P = 2.5). However, TAC exhibits poor aqueous solubility (15–20 µg/mL) [30,31] which may restrict its incorporation into topical formulations and constrain its topical bioavailability [3]. This limitation persists even with conventional enhancement strategies, such as skin occlusion [4] and increased dose or application frequency [5]. Therefore, maintaining maximal thermodynamic activity of TAC throughout the experimental period was considered essential. A TAC solution at 10 mg/mL, assuming near-saturation conditions, was thus employed in this study and was proved to remain detectable in the SC up to 6 h post-application following fractional erbium-doped yttrium aluminium garnet (Er:YAG) laser pretreatment [25]. Our results are also consistent with measurable TAC levels in the SC after 6 h of application, despite using the same low initial TAC loading dose at 83.3 µg/cm^2^ but employing a different enhancement technique involving immediate post-application RFMN treatment. This pilot mechanistic study intentionally employed a single TAC concentration to minimize formulation-related variability and isolate the influence of RFMN parameters on drug delivery enhancement. Alternative formulation strategies, including particulate systems, suspensions, or nanocarrier-based formulations, may exhibit distinct permeation behavior and stability profiles and warrant further investigation.

**Figure 5:**
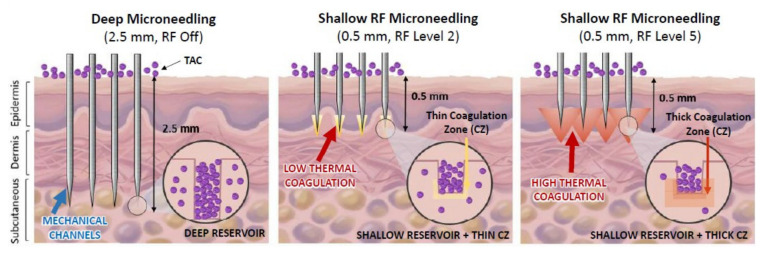
Proposed mechanisms of radiofrequency microneedling (RFMN)-assisted topical triamcinolone acetonide (TAC) delivery.

The degree of drug delivery enhancement has been reported to depend on the RFMN technology and parameter settings [12,16]. The new generation POTENZA™ RFMN device was therefore selected for this study due to its proprietary fusion tip technology utilizing pneumatic pressure to facilitate the penetration of active compounds into the skin along with the adjustable insertion depth (D) raging 0.5–2.5 mm and RF energy settings from 0–1500 mJ, making it particularly suitable for enhancing transdermal drug delivery. This study chose to evaluate the shallowest and deepest insertion depths at 0.5 and 2.5 mm with RF energy levels of 0 (off), 390 mJ (level 2) and 750 mJ (level 5). Any higher RF energy may increase the risk of skin burns. Of these parameter settings, only 2 conditions significantly improved drug permeability: D 2.5 mm + RF 0 which yield the greatest TAC absorption and % TAC absorption (3.5-fold increase compared to TAC application alone) and D 0.5 mm + RF 2 which resulted in a 2.9-fold increase. Other settings at a depth of 2.5 mm also showed slight improvements in drug penetration with increasing RF energy. However, this trend was not observed at a depth of 0.5 mm where drug permeability remained nearly unchanged regardless of RF energy including RF 0 and RF 5. Negligible to undetectable TAC levels were found in the RFMN control group without TAC application and consistent with the previous study using the same quantitative methods [25], thereby confirming the specificity, validity and reproducibility of our quantitative analytical approaches. These findings then pinpoint the importance of parameter settings on drug delivery performance and suggest that the insertion depth is the primary determinant of drug delivery efficacy, likely owing to its direct role in creating deeper mechanical microchannels that bypass the SC barrier [26]. These findings suggest insertion depth is the primary determinant of drug delivery efficacy owing to mechanical microchannel formation bypassing the stratum corneum barrier. RF energy appears to play a secondary role through modulation of local tissue architecture and coagulation characteristics rather than directly altering transport properties of TAC. The observation that the 2.5 mm RF-off condition demonstrated the greatest TAC uptake further supports that mechanical disruption rather than RF thermal energy is the dominant contributor to early penetration of this hydrophobic corticosteroid. Interestingly, this effect becomes more pronounced at shallower insertion depths where limited mechanical disruption reduces intrinsic permeability (Figure 5).

Schematic illustration demonstrating the influence of microneedle insertion depth and RF energy on TAC penetration. Deep microneedling (2.5 mm, RF off) creates extensive mechanical microchannels and a deeper drug reservoir, resulting in the greatest TAC deposition. At shallow insertion depth (0.5 mm), moderate RF energy induces limited thermal coagulation that may enhance drug retention and reser-voir formation, whereas higher RF energy generates a larger coagulation zone that may increase tissue resistance and reduce effective drug diffusion. Overall, TAC delivery is primarily governed by insertion depth, while the contribution of RF en-ergy is parameter-dependent. The schematic illustration was created by the first author (Rosalyn Kupwiwat).

Regarding the underlying mechanisms, the observed effects are likely governed by microchannel depth, the opening period of microchannels and the extent of the coagulation zones (CZs). Our insertion depths of 0.5 and 2.5 mm corresponded to disrupt within the dermis and dermal-subcutaneous layers, respectively. Logically, deeper channels feasibly accommodate a larger volume of the TAC and increase contact area with the surrounding skin, serving as a reservoir for subsequent passive diffusion. Therefore, it is not surprising that our RFMN at a depth of 2.5 mm, regardless of RF energy, overall yielded better outcomes than that of 0.5 mm. This is in agreement with another study using different energy-based modalities (e.g., Er:YAG and CO_2_ lasers) presenting that ablation depth is a key determinant of drug delivery enhancement with greater drug fluorescence intensity observed at 0.3 mm compared to 0.07 mm [32].

The enhancement of topical drug delivery following radiofrequency microneedling (RFMN) is believed to result from multiple complementary mechanisms beyond simple mechanical disruption. Radiofrequency-assisted microporation generates transient aqueous microchannels across the stratum corneum, facilitating drug diffusion while bypassing the primary permeability barrier of the skin [33]. Radiofrequency energy further induces localized thermal effects through ionic oscillation and frictional heating, producing controlled microablation and coagulation that may additionally influence permeant transport dynamics [33]. Fractional microporation techniques have also been shown to increase effective diffusion area and create temporary drug reservoirs within the skin, thereby enhancing penetration into deeper tissue compartments. Confocal imaging studies further demonstrated increased penetration depth and lateral diffusion following microporation, supporting the concept that microchannel formation modifies both the amount and spatial distribution of topically applied compounds. Previous laser-assisted drug delivery literature similarly supports that controlled ablative energy can enhance permeability by generating microscopic transport channels while preserving surrounding untreated tissue, thereby facilitating enhanced penetration with faster recovery compared with conventional ablation techniques [8].

Microchannel persistence also appears to play an important role in delivery efficiency, as prolonged channel patency may permit greater cumulative drug transport [34]. Wang J.V. et al. (2022) reported that neither conventional microneedling nor RFMN at depths of 0.5 and 1 mm produced observable channels by optical coherence tomography immediately after treatment or during follow up to 24 h [12]. Microchannels generated by conventional microneedling without RF energy at shallow depths closed rapidly, whereas insulated RFMN channels were located deeper within the skin and therefore were not readily visualized by optical coherence tomography [12]. Consistent with these findings, Lee W.R. et al. (2026) demonstrated that RFMN-induced microchannels generated at depths of 0.5 and 1 mm with RF energies ranging from 6.1 to 20.4 mJ closed within approximately 12 h based on transepidermal water loss recovery measurements, whereas laser-induced microporation may remain open up to approximately 36 h depending on treatment modality [18]. Perforation and thermal effects were also not clearly appreciable histologically following lower depth (0.5 mm) and lower fluence RFMN treatment [18]. These observations may partly explain our finding that 0.5 mm insertion depth without RF energy did not significantly improve TAC penetration. However, they do not fully explain the RF-associated findings in our study because substantially higher RF energies were used (390 and 750 mJ).

Although treatment at 0.5 mm without RF did not significantly increase cumulative TAC uptake, TAC concentration-depth profiles demonstrated increased superficial deposition together with slightly greater penetration into deeper SC layers compared with TAC application alone, suggesting reservoir formation within the skin. This observation aligns with previous microneedling literature demonstrating increased drug intensity in both superficial and deeper tissue compartments compared with untreated controls [32]. Collectively, these findings suggest that insertion depth and RF energy exert distinct mechanistic influences on TAC delivery enhancement, emphasizing the importance of parameter optimization for device-assisted transdermal corticosteroid delivery. The enhancement of topical drug delivery following radiofrequency microneedling is believed to result from multiple complementary mechanisms beyond simple mechanical disruption. Radiofrequency-assisted microporation generates transient aqueous microchannels across the stratum corneum, facilitating drug diffusion while bypassing the major permeability barrier of the skin [33]. Radiofrequency energy produces localized thermal effects through ionic oscillation and frictional heating within tissue, resulting in controlled microablation and coagulation that may further influence permeant transport dynamics [33].

Fractional microporation techniques have also been shown to increase the effective diffusion area and create temporary drug reservoirs within the skin, thereby enhancing penetration into deeper tissue compartments [18]. Lee et al. demonstrated that radiofrequency microneedling increased drug flux by approximately 5–18-fold compared with passive diffusion, while laser-assisted microporation enhanced skin deposition and transport efficiency depending on parameter selection. Furthermore, confocal imaging confirmed that microporation increased penetration depth and lateral diffusion within surrounding tissue structures, supporting the concept that microchannel formation modifies both the amount and spatial distribution of topically applied agents [18].

Prior laser-assisted drug delivery literature similarly supports that controlled ablative energy can increase permeability by generating microscopic vertical transport channels while preserving surrounding untreated tissue, thereby facilitating enhanced penetration with reduced healing time [8]. Fractional ablative technologies may also permit parameter-dependent modulation of penetration depth, allowing delivery effects to vary according to energy settings and tissue interaction profiles [8].

Importantly, microchannel persistence appears time-dependent. Lee et al. demonstrated closure of radiofrequency microneedling-induced channels within approximately 12 h, whereas laser-generated channels persisted up to approximately 36 h, suggesting that delivery enhancement reflects a dynamic interaction between barrier disruption and skin recovery kinetics [18]. Consistent with these observations, our findings suggest that insertion depth and RF energy may exert distinct mechanistic influences on topical TAC delivery efficiency, supporting parameter optimization as a critical consideration for device-assisted transdermal drug delivery.

Although RFMN requires supervised healthcare delivery and device-associated procedural costs, it provides controlled and reproducible microchannel generation with adjustable treatment parameters integrated into existing dermatologic platforms. Comparative studies evaluating RFMN against alternative enhancement modalities, including microneedle-assisted iontophoresis and other home-based approaches, are warranted to better define relative efficacy, accessibility, and cost-effectiveness [11].

The CZ, a zone of thermally affected skin, was initially considered to act as a barrier to drug diffusion. Nevertheless, recent studies have suggested otherwise that the CZ may be good as a drug reservoir, potentially contributing to sustained drug release [10]. The CZ is influenced by both insertion depth and RF energy. At lower depth and RF energy (0.5 mm/6.1 mJ), the CZ was not detectable but became apparent when increasing depth and RF energy (1 mm/6.1 mJ, 0.5 mm/10.2 mJ, and 0.5 mm/20.4 mJ) [18]. The width of the CZ induced by RFMN has been evaluated to range from 0.06 to 0.1 mm for a needle diameter of 200 µm. Given that our study employed a larger needle diameter (250 µm), greater insertion depths (0.5 and 2.5 mm), and substantially higher RF energy levels (390 and 750 mJ), it is reasonable to infer that the resulting CZ width in our conditions exceeded 0.1 mm.

However, the manner in which drug molecules escape from a CZ-associated reservoir into deeper skin layers depends on the physicochemical properties of the permeant [18]. An absent or minimal CZ (0–0.02 mm) has been reported to favor the diffusion of small lipophilic drugs, likely due to reduced formation of thermal barriers [35]. Conversely, increasing CZ thickness might reduce drug flux from the coagulated region into the surrounding tissue. This can partly explain why TAC, a lipophilic compound, demonstrated only modest or negligible enhancement in our study. At higher RF energy levels, more pronounced coagulation zone (CZ) formation may further impede drug diffusion by increasing tissue densification and reducing effective permeability. At shallow insertion depths, excessive thermal coagulation may disproportionately limit diffusion pathways because limited mechanical disruption cannot sufficiently compensate for the increased resistance associated with tissue coagulation. In addition, greater thermal injury may partially narrow or occlude microchannels, thereby reducing permeability despite successful channel creation. In contrast, moderate superficial thermal exposure may enhance permeation by increasing lipid fluidity and disrupting corneocyte cohesion. These mechanisms may explain why the 0.5 mm/390 mJ condition resulted in significantly greater TAC absorption than the 0.5 mm/750 mJ condition. These findings are further supported by a previous work using fluorescent-labeled polyethylene glycols (PEGs; hydrophilic, MW 350 Da) which demonstrated that CZ thickness significantly influenced dermal uptake and distribution. The PEG uptake was higher in the presence of a thin CZ (0.02 mm) compared to a thicker CZ (0.08 mm) or absence of CZ (0 mm) [10]. Even so, these coagulation zone (CZ)-dependent effects do not fully explain the findings observed at the 2.5 mm insertion depth, where mechanical disruption appeared to play a dominant role in drug delivery enhancement with limited additional benefit from RF energy. At this greater insertion depth, microchannel formation alone likely generated sufficient permeability enhancement by creating larger and more extensive diffusion pathways, thereby reducing the relative influence of RF-associated effects. Under these conditions, RF-induced thermal coagulation may partially counteract the advantages of mechanical channel formation by increasing tissue resistance and impeding diffusion, potentially explaining why TAC permeation was not further enhanced following RF application at the 2.5 mm setting.

In addition to total SC uptake, the TAC concentration versus SC depth profiles revealed that RFMN generally increased TAC levels in the superficial SC layers across all treatment conditions, except the 0.5 mm/750 mJ setting. This suggests that most RFMN parameters facilitated enhanced drug deposition at or near the entry region of the microchannels, consistent with a reservoir-like effect in the upper SC. Moreover, slightly increased TAC levels were detected in deeper SC layers under RFMN conditions compared with TAC application alone, indicating improved permeability beyond the superficial barrier. These findings then suggest that RFMN not only increases overall TAC loading but also modulates its distribution within the SC, potentially forming a reservoir that supports subsequent passive diffusion.

This study has several limitations that should be considered when interpreting the findings. The pilot nature and limited sample size reduce statistical power and may limit external generalizability. However, the intra-individual design minimized inter-subject variability by allowing participants to serve as their own controls. In addition, the quantitative methodology employed in this study was adapted from previously published and validated approaches assessing TAC permeation using tape stripping and UPLC quantification [25]. Larger confirmatory studies are warranted. Drug deposition was assessed exclusively within the SC, which precludes any conclusions regarding penetration or distribution in deeper skin layers. Systemic absorption was not evaluated in the present study because the objective focused on local cutaneous deposition within the stratum corneum. However, the relatively low finite dose used in this study (100 µL of 10 mg/mL TAC solution per treatment site; maximum total dose 8 mg TAC per participant) together with the use of localized treatment areas on healthy skin would be expected to limit systemic exposure. Previous studies reporting pharmacologically significant systemic absorption of topical corticosteroids involved substantially larger doses and markedly impaired skin barrier conditions [35]. Nevertheless, future investigations incorporating systemic pharmacokinetic assessment would further characterize the safety profile of RFMN-assisted corticosteroid delivery. Additionally, assessment at a single predefined time point precluded characterization of dermato-pharmacokinetic profiles, temporal release kinetics, and drug flux. The extent of observed in this study may be limited by the physicochemical properties of the model compound (TAC; hydrophobic) as RFMN generates aqueous microchannels that become rapidly hydrated following perforation [34]. It then preferably facilitates the transport of hydrophilic molecules over hydrophobic compounds [18]. Furthermore, direct comparison with existing RFMN literature remains challenging due to substantial variability in device parameters, physicochemical properties of compounds, application protocols and analytical techniques, making it difficult to interpret the magnitude of the observed effects.

Histologic evaluation was not performed because invasive serial biopsies in healthy volunteers would increase participant burden and introduce potential scarring. Future investigations incorporating histologic assessment or noninvasive imaging modalities may further elucidate tissue remodeling, coagulation zone evolution, and healing dynamics following RFMN treatment.

Further investigations on bioavailability in deeper skin layers (e.g., viable epidermis and dermis), dermato-pharmacokinetics, different drug compounds (both hydrophilic and hydrophobic) and various formulation designs are warranted to comprehensively characterize the cutaneous permeation enhancement capacity and to better understand sustained drug delivery following RFMN treatment. This validated approach is useful to expand dermatologic applications (e.g., alopecia areata, keloids, hypertrophic scars and prurigo nodularis) by overcoming key limitations of conventional topical delivery that commonly depends on lipophilicity and low molecular weight of the drug compounds to achieve therapeutic doses at the dermal target sites while minimizing unnecessary tissue injury and systemic exposure.

## 5. Conclusions

RFMN significantly enhanced topical TAC delivery in human skin, with insertion depth emerging as the primary determinant of delivery enhancement, likely through greater mechanical disruption and microchannel formation that bypass the stratum corneum barrier. In contrast, RF energy exerted a secondary and parameter-dependent effect, with excessive thermal exposure potentially limiting drug permeation through increased tissue coagulation and diffusion resistance. These findings suggest that optimization of insertion depth and RF energy is critical for maximizing RFMN-assisted transdermal delivery. Overall, this study provides quantitative in vivo human evidence supporting evidence-based parameter selection for RFMN-assisted topical therapy and highlights its potential as a targeted transdermal drug delivery platform in clinical dermatology.

## Figures and Tables

**Table 1:** Demographic data of participants in this study (n = 5).

Characteristics		Value
Age (years)	mean ± SD min to max	35.60 ± 8.38 28–48
Gender, n (%)	Female Male	4 (80%) 1 (20%)
Fitzpatrick skin type, n (%)	III IV	1 (20%) 4 (80%)
Skin diseases		None
Other underlying diseases		None
Current medication		None
Allergy		None

## Data Availability

All data generated or analyzed during this study are included in this published article.

## Abbreviations

The following abbreviations are used in this manuscript:

CZ	Coagulation zone
D	Needle depth
Er:YAG	Erbium-doped yttrium aluminum garnet
MN	Microneedling
OCT	Optical coherence tomography
PDA	Photodiode array
PEG	Polyethylene glycol
RF	Radiofrequency
RFMN	Radiofrequency microneedling
SC	Stratum corneum
SD	Standard deviation
SIRB	Siriraj Institutional Review Board
TAC	Triamcinolone acetonide
TCTR	Thai Clinical Trials Registry
TEWL	Transepidermal water loss
UPLC-PDA	Ultra-performance liquid chromatography with photodiode array detection
VAS	Visual analog scale

## References

[1] Nino M., Calabrò G., Santoianni P. (2010). Topical delivery of active principles: The field of dermatological research. Dermatol. Online J..

[2] Gorzelanny C., Mess C., Schneider S.W., Huck V., Brandner J.M. (2020). Skin barriers in dermal drug delivery: Which barriers have to be overcome and how can we measure them?. Pharmaceutics.

[3] Malkinson F.D., Kirschenbaum M.B. (1963). Percutaneous absorption of C14-labeled triamcinolone acetonide. Arch. Dermatol..

[4] Pellanda C., Strub C., Figueiredo V., Rufli T., Imanidis G., Surber C. (2007). Topical bioavailability of triamcinolone acetonide: Effect of occlusion. Ski. Pharmacol. Physiol..

[5] Pellanda C., Ottiker E., Strub C., Figueiredo V., Rufli T., Imanidis G., Surber C. (2006). Topical bioavailability of triamcinolone acetonide: Effect of dose and application frequency. Arch. Dermatol. Res..

[6] Hengge U.R., Ruzicka T., Schwartz R.A., Cork M.J. (2006). Adverse effects of topical glucocorticosteroids. J. Am. Acad. Dermatol..

[7] Tatu A.L., Ionescu M.A., Nwabudike L.C. (2018). Contact allergy to topical mometasone furoate confirmed by rechallenge and patch test. Am. J. Ther..

[8] Sklar L.R., Burnett C.T., Waibel J.S., Moy R.L., Ozog D.M. (2014). Laser assisted drug delivery: A review of an evolving technology. Lasers Surg. Med..

[9] Lim H.K., Jeong K.H., Kim N.I., Shin M.K. (2014). Nonablative fractional laser as a tool to facilitate skin penetration of 5-aminolaevulinic acid with minimal skin disruption: A preliminary study. Br. J. Dermatol..

[10] Haak C.S., Hannibal J., Paasch U., Anderson R.R., Haedersdal M. (2017). Laser-induced thermal coagulation enhances skin uptake of topically applied compounds. Lasers Surg. Med..

[11] Arshad M.S., Hussain S., Zafar S., Rana S.J., Chohan T.A., Hamza M., Ahmad Z. (2024). Transcutaneous delivery of dexamethasone sodium phosphate via microneedle-assisted iontophoretic enhancement: A potential therapeutic option for inflammatory disorders. Pharm. Res..

[12] Wang J.V., Mehrabi J.N., Zachary C.B., Geronemus R.G. (2022). Evaluation of device-based cutaneous channels using optical coherence tomography: Impact for topical drug delivery. Dermatol. Surg..

[13] Meesters A.A., Nieboer M.J., Kezic S., de Rie M.A., Wolkerstorfer A. (2018). Parameters in fractional laser assisted delivery of topical anesthetics: Role of laser type and laser settings. Lasers Surg. Med..

[14] Taudorf E.H., Lerche C.M., Erlendsson A.M., Philipsen P.A., Hansen S.H., Janfelt C., Haedersdal M. (2016). Fractional laser-assisted drug delivery: Laser channel depth influences biodistribution and skin deposition of methotrexate. Lasers Surg. Med..

[15] Haak C.S., Bhayana B., Farinelli W.A., Anderson R.R., Haedersdal M. (2012). The impact of treatment density and molecular weight for fractional laser-assisted drug delivery. J. Control. Release.

[16] Seiger K., Driscoll W., Messele F., Golbari N.M., Fan X., Holmes J., Zachary C.B. (2024). Use of optical coherence tomography to assess properties of cutaneous defects following radiofrequency microneedling and laser treatment. Lasers Surg. Med..

[17] Magro I., Kochhar A., Arnaoutakis D., Karimi K. (2022). Transcutaneous radiofrequency microneedling in the facial plastic surgeon’s practice: A review. Facial Plast. Surg. Aesthet. Med..

[18] Lee W.R., Alalaiwe A., Tsai M.T., Hu S., Chang F.H., Chien M.Y., Sung H.-C., Fang J.-Y. (2026). Fractional microporation-guided delivery of nanoencapsulated drugs for enhanced cutaneous and follicular absorption: A comparison of ablative laser and radiofrequency microneedling. Drug Deliv. Transl. Res..

[19] Yu A.J., Luo Y.J., Xu X.G., Bao L.L., Tian T., Li Z.X., Dong Y.-X., Li Y.-H. (2018). A pilot split-scalp study of combined fractional radiofrequency microneedling and 5% topical minoxidil in treating male pattern hair loss. Clin. Exp. Dermatol..

[20] Yogya Y., Wanitphakdeedecha R., Wongdama S., Nanchaipruek Y., Yan C., Rakchart S. (2022). Efficacy and safety of using noninsulated microneedle radiofrequency alone versus in combination with polynucleotides for treatment of periorbital wrinkles. Dermatol. Ther..

[21] Lyu J., Jiang L., Tang M., Zheng L., Liu S. (2024). Microneedle fractional radiofrequency associated with drug delivery for facial atrophic acne scars and skin rejuvenation. J. Cosmet. Laser Ther..

[22] Akerman L., Kaplan B., Mimouni D., Nosrati A., Solomon-Cohen E. (2025). Topical tranexamic acid-mediated with non-insulated microneedling radiofrequency for the treatment of melasma. Isr. Med. Assoc. J..

[23] Kupwiwat R., Techapichetvanich T., Bhorntarakcharoen W., Sittiwanaruk S., Li J.B., Manuskiatti W. (2026). Transdermal delivery of poly-L-lactic acid via fractional microneedle radiofrequency for atrophic acne scars: A split-face randomized study in Fitzpatrick skin types III to V. Dermatol. Ther..

[24] Ekelem C., Thomas L., Van Hal M., Valdebran M., Lotfizadeh A., Mlynek K., Mesinkovska N.A. (2019). Radiofrequency therapy and noncosmetic cutaneous conditions. Dermatol. Surg..

[25] Thitilertdecha P., Wannawittayapa T., Buranaporn P., Rejuso-Kalbit C., Kupwiwat R., Poungpairoj P., Tantithavorn V., Onlamoon N., Manuskiatti W. (2025). Fractional erbium-doped yttrium aluminum garnet laser-assisted drug delivery: Impact of triamcinolone acetonide formulation on drug permeation. Drug Deliv. Transl. Res..

[26] Russell L.M., Wiedersberg S., Delgado-Charro M.B. (2008). The determination of stratum corneum thickness: An alternative approach. Eur. J. Pharm. Biopharm..

[27] Thitilertdecha P., Rowan M.G., Guy R.H. (2015). Topical formulation and dermal delivery of active phenolic compounds in the Thai medicinal plant *Clerodendrum petasites* S. Moore. Int. J. Pharm..

[28] Wongman A., Tummarintra P., Thitilertdecha P., Boonprasert R. (2021). Development and validation of an ultra performance liquid chromatography photodiode array method for quantification of low levels of triamcinolone acetonide in skin permeation studies. J. Basic Appl. Pharmacol..

[29] Singh I., Morris A.P. (2011). Performance of transdermal therapeutic systems: Effects of biological factors. Int. J. Pharm. Investig..

[30] Behl C.R., Block L.H., Borke M.L. (1976). Aqueous solubility of 14C-triamcinolone acetonide. J. Pharm. Sci..

[31] Block L.H., Patel R.N. (1973). Solubility and dissolution of triamcinolone acetonide. J. Pharm. Sci..

[32] Nieboer M.J., Meesters A.A., Almasian M., Georgiou G., de Rie M.A., Verdaasdonk R.M., Wolkerstorfer A. (2020). Enhanced topical cutaneous delivery of indocyanine green after various pretreatment regimens: Comparison of fractional CO_2_ laser, fractional Er:YAG laser, microneedling, and radiofrequency. Lasers Med. Sci..

[33] Sintov A.C., Krymberk I., Daniel D., Hannan T., Sohn Z., Levin G. (2003). Radiofrequency-driven skin microchanneling as a new way for electrically assisted transdermal delivery of hydrophilic drugs. J. Control. Release.

[34] Banzhaf C.A., Ortner V.K., Philipsen P.A., Haedersdal M. (2019). The ablative fractional coagulation zone influences skin fluorescence intensities of topically applied test molecules: An in vitro study with fluorescence microscopy and fluorescence confocal microscopy. Lasers Surg. Med..

[35] Aalto-Korte K., Turpeinen M. (1995). Quantifying systemic absorption of topical hydrocortisone in erythroderma. Br. J. Dermatol..

